# Remodeling an existing rare disease registry to be used in regulatory context: Lessons learned and recommendations

**DOI:** 10.3389/fphar.2022.966081

**Published:** 2022-09-23

**Authors:** Marina Mordenti, Manila Boarini, Fabio D’Alessandro, Elena Pedrini, Manuela Locatelli, Luca Sangiorgi

**Affiliations:** Department of Rare Skeletal Disorders, IRCCS Istituto Ortopedico Rizzoli, Bologna, Italy

**Keywords:** disease registry, real-world evidence (RWE), orphan drug, rare diseases, multiple osteochondromas, EMA initiative

## Abstract

Disease registries have been used as an interesting source of real-world data for supporting regulatory decision-making. In fact, drug studies based on registries cover pre-approval investigation, registry randomized clinical trials, and post-authorization studies. This opportunity has been investigated particularly for rare diseases—conditions affecting a small number of individuals worldwide—that represent a peculiar scenario. Several guidelines, concepts, suggestions, and laws are already available to support the design or improvement of a rare disease registry, opening the way for implementation of a registry capable of managing regulatory purposes. The present study aims to highlight the key stages performed for remodeling the existing Registry of Multiple Osteochondromas—REM into a tool consistent with EMA observations and recommendations, as well as to lead the readers through the entire adapting, remodeling, and optimizing process. The process included a variety of procedures that can be summarized into three closely related categories: semantic interoperability, data quality, and governance. At first, we strengthened interoperability within the REM registry by integrating ontologies and standards for proper data collection, in accordance with FAIR principles. Second, to increase data quality, we added additional parameters and domains and double-checked to limit human error to a bare minimum. Finally, we established two-level governance that has increased the visibility for the scientific community and for patients and carers. In conclusion, our remodeled REM registry fits with most of the scientific community’s needs and indications, as well as the best techniques for providing real-world evidence for regulatory aspects.

## Introduction

Real-world evidence (RWE) is a widely used definition for evidence on health and healthcare gathered from several sources. In fact, RWEs are the results of analyses on real-world data (RWD), commonly defined as “routinely collected data of a patient’s health status or delivery of health care from a variety of sources.” This term refers to a large and constantly expanding set of data that is beyond clinical trials, including, but not limited to, information captured during clinical settings, patient-generated data, hospital databases, and disease registries, as well as data collected by wearable and personal devices, such as a smartwatch and mobile applications (Sherman et al., 2016; Makady et al., 2017; Bolislis et al., 2020). These data are considered an intriguing way to explore the rare disease scenario and, subsequently, to investigate orphan drugs (Wu et al., 2020; Crisafulli et al., 2019).

Rare diseases (RDs) are a heterogeneous group of conditions that affect a limited number of individuals, with a prevalence defined in Europe as less than 5:10000. Nevertheless, RDs impair 36 million people in Europe and 300 million people globally, so affecting 4% of the total world population, and most of them—about 70%—begin the course during childhood (Nguengang Wakapet et al., 2020; Rare diseases, 2022). RDs are frequently characterized by a delay in diagnosis; they are understudied and neglected on a general basis and, even more, for treatments and therapies since pharmaceutical companies are reluctant to support research and development of dedicated medicines, named orphan drugs (Schuller et al., 2018).

Orphan drugs or orphan medical products (OMPs) were first defined and regulated in the Orphan Drugs Act in 1983 by the United States. At the European level, OMPs are defined as a medicine for the diagnosis, prevention, or treatment of a life-threatening or chronically debilitating condition that is rare or where the medicine is unlikely to generate sufficient profit to justify research and development costs [EMA]. The European Parliament adopted, in 2000, the Regulation (EC) No 141/2000—the Orphan Regulation—after its publication in the Official Journal of the European Communities (European Communities, 2000). This regulation describes the procedures for the designation of orphan medicine; establishes a dedicated committee, the COMP (Committee for Orphan Medicinal Products); and delineates the incentives for the development and placing on the market of OMPs. In fact, considering that OMPs are characterized by no profitability, some financial government incentives are required (Llinares, 2010; Auvin et al., 2018; Postma et al., 2022; Schuller et al., 2018).

RWDs collected within disease registries have a key role in several processes related to increasing the knowledge of RDs (Zaletel et al., 2015; Hollak et al., 2020). Disease registries are defined as “organized systems that use observational methods to collect uniform data on a population defined by a particular disease, condition, or exposure, and that is followed over time” (Gliklich et al., 2020). Disease registries are crucial for boosting natural history studies for understanding disease evolution and prediction of severity research, for promoting epidemiological investigation, for developing guidelines and recommendations, and for evaluating the impact of treatments, both in terms of collecting clinical trial data before regulatory approval and supporting post-marketing authorization (Parums, 2021; Hollak et al., 2020; Kolker et al., 2022). Although the disease registries have been developed primarily for observational research, their role as multi-purpose instruments has been widely recognized (Kolker et al., 2022), and to date, the number of interventional studies nested in a registry, such as the registry-based randomized trial (RRCT) design, is increasing (James et al., 2015; Jansen-van der Weide et al., 2018; Kolker et al., 2022).

This has also been highlighted by the European Medicines Agency (EMA), which, in 2015, launched the Patient Registries Initiative, focused on promoting use of existing registries in collecting information for contributing regulatory assessments, particularly for post-authorization safety study (PASS) and post-authorization effectiveness study (PAES) [McGettigan et al., 2019], and on providing methodologies for establishing new registries. Therefore, the availabilities of well-structured registries have already had a recognized role in the drug authorizations process as outlined by the Myozyme example, an OMP approved by the EMA for Pompe disease and for which a patient registry played a key role in the risk–benefit evaluation (Byrne et al., 2011; EMA, 2018).

We aim to present the main steps for remodeling the existing Registry of Multiple Osteochondromas (REM-NCT04133285) as a tool compliant with EMA observations and recommendations and guide the readers along the entire adapting, remodeling, and optimizing process.

## Assessment of guidelines, principles, and regulations

We performed an evaluation of the main handbooks, guidelines, principles, directives, and recommendations available for registry setup or remodeling; in parallel, we have accurately taken into consideration the EMA initiative of expanding the disease registries as a system for supporting OMP research projects, preclinical studies, and/or post-authorization monitoring. These documentations paved the way for the entire tailoring process: assisting the transformation of a disease registry already in place to a tool that pursues regulatory purposes. Above the multiple disparate sources of information available on registry establishment and development, the following tools represented the most useful, freely available, and widely recognized supports.

### Registries for evaluating patient outcomes: a user’s guide

The Agency for Healthcare Research and Quality (AHRQ) has drafted a reference handbook aiming to provide best practices to guide, plan, design, manage, analyze, and evaluate registries, named “Registries for Evaluating Patient Outcomes: A User’s Guide” in 2007. This guide has been revised several times following the evolutionary nature of the registries and aiming to integrate all learned lessons from the technical and cultural point of view. The current version, released in 2020, is the fourth and gives additional advice on 1) collaboration with patients (and patients’ associations) across the registry lifecycle, 2) the crucial role of data standards, 3) reusing of existing data sources, 4) ethical and legal aspects, and 5) increasing interest in using registries as sources of RWD/RWE for informing decision-making. (Gliklich et al., 2020).

### The PARENT joint action

The Join Action (JA) entitled “cross-border PAtient REgistries iNiTiative—PARENT” under the EU’s Health Programme 2008–2013 was created with the purpose of helping the European countries in developing patient registries in peculiar fields (i.e., RDs) and rationalize and harmonize the development and governance of registries, facilitating analyses of secondary data for public health and research purposes.

Within this JA has been drafted the “methodological guidelines and recommendations for efficient and rational governance of patient registries,” a complex document that provides guidance and instruments, on an EU level, to set up (or remodel) a patient registry and oversee it, to increase interoperability and data exchange among registries, and to facilitate analyses of secondary data for public health and research purposes [available at: https://ec.europa.eu/health/system/files/2016-11/patient_registries_guidelines_en_0.pdf Accessed: 10 May 2022].

### FAIR guiding principles

The FAIR principles, signifying Findability, Accessibility, Interoperability, and reuse of data, represent a set of guidelines that enables and simplifies data sharing from multiple sources [Wilkinson et al., 2016; available at: https://www.go-fair.org/Accessed: 10 May 2022]. The FAIR concept has been vastly suggested by the scientific community as a tool for enabling data collection in disease registries and has become a recommended approach, particularly in rare disease scenarios (Zare Jeddi et al., 2021; Kodra et al., 2018; Kölker et al., 2022). In fact, the FAIR approach aims to extensively expand registry data usage in the most efficient way, particularly for the benefit of RD patients (Kodra et al., 2018).

### Legal regulations

In Europe, the main privacy-preserving set of rules is summarized in the General Data Protection Regulation—GDPR [EU Regulation 2016/679. Available at: https://eur-lex.europa.eu/eli/reg/2016/679/oj Accessed: 10 May 2022]. This regulation, put into effect in May 2018, aims to ensure natural person rights, albeit allowing open movement of data and pursuing a careful balancing act between protection to prevent misuse and potential accessibility for research and networking purposes. The GDPR has been implemented, modified, and integrated at the national level, with additional laws and legislations.

### The EMA initiative

The European Medicine Agency has launched, in September 2015, an initiative for patient registries, aiming to discover new ways to increase the role and use of existing disease registries [Initiative for patient registries. Available at: http://www.ema.europa.eu/docs/en_GB/document_library/Other/2015/10/WC500195576.pdf Accessed: 10 May 2022], highlighting their potential impact in benefit–risk evaluation of medicinal products like OMPs. In the following years, this initiative gained wide consensus, and additional supporting materials have been published by the EMA [available at: https://www.ema.europa.eu/en/guideline-registry-based-studies Accessed: 10 May 2022] and by the scientific community (McGettigan et al., 2019; Jonker et al., 2017; Pacurariu et al., 2018). In particular, three themes have arisen from expert stakeholders’ consultation as the factors that assist the progress of using a registry for regulatory assessment: 1) the nature of data and process quality, 2) governance and ethical and legal issues, and 3) stakeholder communication and benefit-risk assessment (McGettigan et al., 2019).

### The REM—Registry of Multiple Osteochondromas (NCT04133285)

Since 1950, our institution has treated multiple osteochondromas and has acquired a high volume of data on this condition. The first attempt to organize these data was in 2003, when a dedicated data collection was enacted, leading in 2013 to the establishment of the Registry of Multiple Osteochondromas (REM) approved by the institutional review board (IRB). During the REM’s 10-year history, A.C.A.R. Onlus, the Patient Association of Multiple Osteochondromas and Enchondromatoses, engaged in and sponsored several activities of the REM (i.e., Advisory Board).

**FIGURE 1:**
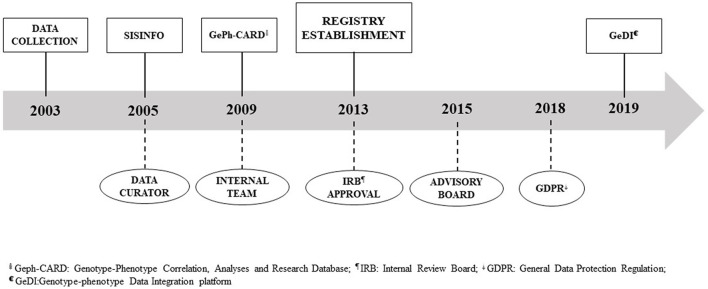
REM registry timeline.

Various tools have been developed throughout time to capture patient data in a systematic manner, ranging from a locally installed access-based application called SISINFO to a web application named GeDI. Similarly, the organization and governance evolved over the last 20 years. The registry progress is reported in Figure 1.

To date, the REM collects a structured dataset containing vital information regarding patient demographics, disease onset, comorbidities, genetic data, family history, and clinical anomalies, with particular attention to treatment details and intermediate outcomes, in accordance with national and international regulations (GDPR). All of this is recorded using standard coding terminologies, regulatory recommendations, and international guidelines. According to the legislation in force, the patient participant in the REM registry is the primary owner of the data, with the Istituto Ortopedico Rizzoli acting as the data controller.

## Results and recommendations

To face and solve the challenges of remodeling an existing registry, having the opportunity to realize a tool capable of collecting RWD and other information for regulatory purposes, we have taken into consideration several methodological aspects and have designed linked activities and implementations.

The following sections are based on our experience in remodeling the REM Registry first implemented for natural history studies, epidemiological research, and genotype–phenotype correlation into a tool for supporting regulatory assessment. The steps are intended to support clinicians, researchers, and other potential stakeholders in this process, applying the most pertinent and up-to-date guidelines and principles and taking into consideration the existing European legislation. Therefore, those indications are not mandatory and represent an experienced suggestion.

### Implementation of standards and ontologies

A major challenge in data collection for subsequent integration and exchange is semantic interoperability (de Mello et al., 2022). The tailoring of a registry for regulatory purposes requires a proper ecosystem of vocabularies, taxonomies, and standardized values—recognized by the scientific community—for clinical annotations, genetic background, RWD, and other information. This interoperability is centered on specific data elements, dedicated ontologies, and general common terminologies.

In the RD scenario, the “set of common data elements for Rare Disease Registration” is the tool released by the EU RD platform to practically support interoperability [available at: https://eu-rd-platform.jrc.ec.europa.eu/set-of-common-data-elements_en accessed: 10 May 2022]. It gives an indication for collection of 16 core elements, arranged in eight groups, covering general information, from personal data to a brief overview of patients’ diseases. This instrument has been designed for coding the data format (i.e., date: dd/mm/yyyy) and recommending proper ontologies and standards (i.e., Orpha Code). With the aim of pursuing semantic interoperability, and giving a registry the role of supporting regulatory assessment, we strongly advise individuating best ontologies. Our practice with the REM suggests applying the set of common data element coding tools: 1) disease: Orpha Code from ORPHANET (or ICD-9/10), 2) genetic diagnosis: International Classification of Mutations (HGVS/HGNC), 3) genotyping: Human Phenotype Ontology (HPO), and 4) disability: International Classification of Functioning and Disability (ICF). In addition, to support the collection of all the other information, we have individuated additional ontologies and terminologies like Medical Dictionary for Regulatory Activities (MedDRA) or Logical Observation Identifiers Names and Codes (LOINC). Selected ontologies and nomenclatures are listed in Table 1.

**TABLE 1:** Selected standards used in the REM Registry.

Name	Acronym	Main topic/area	Website
Systematized Nomenclature in medicine—clinical Term	SNOMED-CT	Disease nomenclature	http://www.nlm.nih.gov/research/umls/Snomed/snomed_main.html
ORPHANET Nomenclature	ORPHANET	Disorder nomenclature	https://www.orpha.net/consor/cgi-bin/index.php?lng=EN - http://www.orphadata.org/cgi-bin/ORPHAnomenclature.html
Human Phenotype Ontology	HPO	Standardized vocabulary	https://hpo.jax.org/app/
HUGO Gene Nomenclature Committee	HGNC	Genomic nomenclature	https://www.genenames.org/
Medical Dictionary for Regulatory Activities	MedDRA	Dictionary	https://www.meddra.org/
Chemical Entities of Biological Interest Ontology	ChEBI	Dictionary	https://www.ebi.ac.uk/chebi/
Non-Pharmacological Interventions	NPIs	Non-pharmacological interventions nomenclature	https://bioportal.bioontology.org/ontologies/NPI/?p=summary
Logical Observation Identifier Names and Codes	LOINC	Standardized vocabulary	https://loinc.org/
Ontology for Biomedical Investigations	OBI	Ontology	http://obi-ontology.org/
Open Biological and Biomedical Ontology Foundry	OBO	Ontology	https://obofoundry.org/resources

Interoperability is a core principle of the FAIRification process that also encompasses the findability, accessibility, and reusability of data for humans and machines. The integration of FAIR principles in the RD registry is a very powerful action, making data available for a wider community and broader use and avoiding re-collection of data. On the other hand, FAIR is a complex and demanding process, requiring highly specific competencies from data stewardship to legal aspects and disease expertise in addition to registry management and data curation. In this perspective, we have embarked on a path aiming to pursue the FAIR guidance as much as possible, considering this action as a continuously ongoing process to improve data integration.

### Data quality and reliability

In addition to the mentioned set of common data elements for RD registration, there is no unique and perfect list of elements to be collected. Different diseases shall require different data. In our experience, we have individuated several data domains: 1) personal information, 2) diagnosis, 3) clinical data, 4) family data, 5) genetic information, 6) surgery, 7) lab test, 8) functional assessment, 9) quality of life, and 10) drug therapy and treatment. Each of these sections has been designed to support clinicians, geneticists, and researchers in tracking all the aspects of the patients and disease.

As shown in Table 2, the inclusion of new parameters for regulatory purposes has notably increased the captured features, changing the overall numbers from 147 to 190 (almost 30%).

**TABLE 2:** Comparison between already-in-place domains and parameters versus new domains and parameters designed for RWE capturing in regulatory context.

Domain	Already in place		Implemented for regulatory context		Total no. of parameters
	Present	No. of parameters	Present	No. of new parameters	
Personal data	✓	29	✓	—	29
Diagnosis	✓	16	✓	—	16
Clinical data	✓	59	✓	12	71
Family data	✓	4	✓	—	4
Genetics	✓	32	✓	—	32
Surgery	✓	7	✓	1	8
Lab test		—	✓	8	8
Functional assessment		—	✓	6	6
Quality of life		—	✓	6	6
Drug therapy and treatments		—	✓	10	10
All domains		147		43	190

To assure the reliability of collected information—a pre-requisite for disease registries—we have put in place several strategic actions. First, data capturing is carried out by experts of registry staff, primarily data curators and data managers. Second, the data sources must be highly trustworthy. Accordingly, we have decided to collect information derived from health records, medical reports, and other clinical documentation (i.e., radiological reports). Moreover, a double-check is always in place for captured data to minimize human error. Finally, collecting longitudinal data and following up with patients offers the opportunity to further validate the information.

Data quality is mainly defined by three attributes: consistency (uniformity of data over time), accuracy (no errors, contradictions, and duplicates), and completeness (proportion of missing data and absence of core variables) (McGettigan et al., 2019). We have pursued those aspects in the REM registry from the beginning, and we are continuously optimizing them, particularly from a regulatory perspective; in fact, defined standards, in terms of ontologies as well as working procedures have been put in place.

Sections have been designed to guide the users in data collection, including several mandatory fields and implementing as many drop-down menus and lookup forms as possible. In addition, the IT platform has some internal checks for data inconsistency and alerts for missing documentation (i.e., informed consent).

### Governance

According to “methodological guidelines and recommendations for efficient and rational governance of patient registries” and “Registries for Evaluating Patient Outcomes: A User’s Guide,” governance is an organizational foundation of registries that primarily encompasses the guidance and decision-making through key activities: building the team, defining overall direction and objectives, identifying stakeholders, promoting layman and scientific dissemination, facing ethical, legal, and social issues (ELSI), and planning registry sustainability.

In 2013, at the establishment of the REM registry, following IRB REM approval, we ameliorated the internal team, including several competencies to better take care of data management and curation, to update all the tools (information and communication technologies –(ICT) platform, procedures, and policies ), to train registry users, and to network with institutional boards (Figure 1). Two years later, an Advisory Board (AB) has been constituted to deal with ELSI issues to increase patient engagement and involvement of other stakeholders, to evaluate past activities, and to define future directions. The AB comprised multiple competencies—including, but not limited to, bioethics, patients’ perspective, privacy, quality, regulatory framework, and ICT—and is shared with the biobank of genetic samples (BIOGEN) since opportunities and challenges in registry and biobanking are analogous.

This two-level governance has been properly created from the beginning, addressing regulatory aspects, and has driven several activities to increase visibility and dissemination, support registry sustainability, and increase patients’ engagement and data access. In fact, the submission of the disease registries to the ClincialTrial.gov portal has very positively impacted on visibility and enrollment. In the last 4 months (February—May 2022), we have received 23 contacts of interest (20 of them from the United States) in participating by patients and carers from around the world. Similarly, the REM registry has been included on the European Platform on Rare Disease Registration (ERDRI.dor) and on the online directory of existing rare disease databases, registries, and biobanks named “Registry and Biobank Finder” created by the RD-CONNECT project.

The increased visibility of the REM has raised some data access issues, requiring the definition and adoption of a data access policy (DAP) model. At present, the primary needs and requirements for data access have been identified, and the DAP is under drafting. This policy specifies internal criteria for access to registry data and guarantees that data access is in compliance with national and international regulations in order to give broad and efficient access to data. It defines and validates data access for different stakeholders with regard to 1) patients' consent specification, 2) relevant and legitimate research purpose, 3) type of stakeholders (i.e., profit vs no profit), 4) data access agreement, and 5) data aggregation. Furthermore, for ultra-rare diseases or very small sub-cohorts of patients, extra steps will be taken to avoid patient identification. The REM registry itself and the research derived from collected data have been promoted to the scientific and patient community *via* poster and oral presentations and through scientific and layman publications and articles. All those activities have opened new opportunities and projects at the national and international levels, impacting on visibility and sustainability. This latter topic, which also includes fund-raising activities, is a vast and complex challenge for long-term maintenance of registries. The REM registry, capturing traceable and structured data, was noticed by the Clementia Pharmaceuticals company (acquired by IPSEN Pharma), which funded analyses and elaborations for investigating the natural history and disease evolution of the disease (Mordenti et al., 2020), with the intent to draft the protocol for repurposing of an orphan drug.

Finally, the governance had taken care of training activities for registry staff (particularly for new personnel) and for data collectors. To provide knowledge on additional registry integrations derived from remodeling activities, we have organized ad hoc dedicated training for all the end users. In addition, new staff has been extensively trained, and dedicated materials and user guides are always available to guide data capturing and contributions.

## Discussion and lessons learned

Disease registries have been acknowledged as the par excellence instrument for the collection of real-world data to perform natural history studies and epidemiological assessments. More recently, they have been recognized as valuable sources of data for supporting regulatory aspects, particularly in the RD scenario. In fact, from a methodological perspective, disease registries can support the development of clinical protocols, can assist the conduction of RRCTs, and can evaluate treatment efficacy and effectiveness.

Registries, in our experience, can be used as a source of information during the drafting of the clinical protocol. This information can assist on several levels: in the definition of the study population, in the identification of the inclusion and exclusion criteria, in the baseline (and disease evolution) data collection, and in the individuation of eligible patients (Mordenti et al., 2020). Furthermore, the registries may support the estimation of the nuisance parameter rate, making the sample size more appropriate (Nikolakopoulos et al., 2014). For instance, the authorization of Myozyme, approved by the EMA for Pompe disease with infantile-onset, was based on a cohort of untreated patients furnished by a registry (EMA, 2018). In fact, in ultra-rare diseases, the registries can contribute with a historical control cohort to conduct a non-randomized trial. Nonetheless, this approach should be limited due to the high risk of biases (Franklin et al., 2022).

Recently, registries have been considered for treatment evaluation studies such as the RRCT. This new research method is based on two main registry features: the presence of high-quality data and availability of potentially eligible participants. If used in conjunction with a randomization tool, the registry can serve as a trial database substitute. This approach is potentially characterized by reduction in costs, increased generalizability of findings, fast recruitment (due to the less restrictive inclusion and exclusion criteria), and an almost complete overview of the reference population (Li et al., 2016). Nonetheless, RRCT seems to face similar biases that are present in randomized controlled trials (James et al., 2015). Therefore, taking into consideration the mentioned features, RRCT can be enlisted as a pragmatic trial, giving information on its effectiveness in a real-world setting (Dal-Ré et al., 2018; Thorpe et al., 2009).

Finally, post-authorization studies such as PASS and PAES could largely benefit from registry usage, particularly in RDs. In fact, existing registries, providing a longitudinal long-term collection of RWD, guide the users in assessing the risk of rare adverse events and properly addressing safety concerns (Hollak et al., 2015). In addition, disease registries capture data regarding all medications used by each patient, allowing future comparisons (Jonker et al., 2017).

The role of registries in regulatory assessment has been widely investigated and promoted by the scientific community (Hollak et al., 2015; Hollak et al., 2020) and by the EMA and its Patient Registries Initiative, aiming to create a registry framework that involves all stakeholders (registry holders and staff, patient associations, regulatory agencies, and pharmaceuticals companies) (McGettigan et al., 2019; Jonker et al., 2021; Jonker et al., 2017).

Following the mentioned results, up-to-date guidelines, and recommendations, we have remodeled the REM registry into a tool capable of capturing data for regulatory decision-making.

The incorporation of ontologies has been considered a mandatory aspect and, consequentially, we have defined these to implement dedicated standards to register regulatory data. The selected terminologies (i.e., MedDRA) are recognized worldwide and represent the reference for drugs, devices, and side effects (Rath et al., 2012). Additionally, we have also implemented less-used ontologies (i.e., NPIs) since, in our experience, they represented a precious source of standardization for peculiar niche topics. We have overall implemented the registered parameters of almost 30%, and these supplemental ontologies were required to properly describe the features of the disease collected on REM.

This increased content has had, at present, multiple impacts that have both positively and negatively affected the work of registry staff. We have recorded a more time-consuming effort in data collection and training activities—both as teachers and as auditors, depending on competencies. Furthermore, this implementation has had a notable impact on economic aspects and dedicated time due to platform remodeling and subsequent testing phase, before the effective go live. On the other hand, the mentioned criticalities have been paid off by an increase in visibility at the national, European, and international levels, opening to new networking activities and projects, emphasizing the registry attractiveness for pharmaceutical and medical device companies (i.e., Clementia Pharmaceuticals), and renovating the engagement of patient associations.

We have gained a higher level of attractiveness for patients due to the dissemination activities put in place by the two-level governance. The worldwide interest manifested by patients and patients' families in participating in the registry is a valuable result for rare disease scenario that is frequently characterized by data scattering and a very small dataset, limiting epidemiological studies and clinical research.

Despite this, we faced some criticalities that at present are still open issues. The most challenging point has been the hardship of tailoring a registry capable of properly managing all the mentioned purposes. This issue needs a continuous balance between the flexibility required by new opportunities and the mandatory rigidity in data registering.

In addition, we shall highlight the time-consuming and cost-intensive effort to keep data up to date, not only in following up on patient's information but mostly due to new scientific findings (i.e., changing the clinical significance of a genetic variant) or standard reassessment (i.e., change in disease classification). Appropriate budgeting for registry staff is essential. The registry requires a unique blend of skills, including registry manager, data curator, clinician, administrative staff, epidemiologist, and biostatistician, and extra positions based on registry specificities. These personnel, even if allocated on a temporary basis to registry activities, need to be continuously trained on topics related to new regulations and innovative scientific findings. Additionally, funding and resources are fundamental for data storage, platform implementation, or (re)modeling for regulatory purposes and for all infrastructure maintenance aspects. In our experience, long-term sustainability can be ensured with the support of patients’ organizations that can co-fund specific tailoring of infrastructure and staff salary. Moreover, regional, national, and international grants can support registry activities or personnel as part of research projects. Last, investor stakeholders, like pharma- or medical device company or private sources at large, can compensate for part of the cost. Since registry costs are frequently very consistent, a multi-supporter approach represents, in our experience and in literature, the most secure way for long-term sustainability, limiting the risk of lack of funding (Kodra et al., 2018). In conclusion, our remodeled REM registry meets almost all the features, requirements, and indications highlighted by the scientific community as the best practices or the most adequate approaches for producing RWE for regulatory aspects.
